# Costs of Formal and Informal Home Care and Quality of Life for Patients with Multiple Sclerosis in Sweden

**DOI:** 10.1155/2014/529878

**Published:** 2014-03-04

**Authors:** Marianne Svensson, Liberty Fajutrao

**Affiliations:** ^1^The Swedish Institute for Health Economics, Box 2127, 220 02 Lund, Sweden; ^2^Merck Serono Sweden, Merck AB, Box 3033, 169 03 Solna, Sweden

## Abstract

Disease progression in multiple sclerosis leads to dramatic changes in a person's ability to perform daily activities and increases reliance on external help. This study aims to describe and to estimate costs of formal/informal home care and quality of life related to multiple sclerosis. A mailed survey to a random sample of MS sufferers (*n* = 1500) collected data on the number of hours of home care received, type of help, productivity losses, quality of life, and disease characteristics. Costs for home care were estimated in 2012 € and factors that may influence the likelihood of getting home care were also evaluated. Formal care was given to 27% of the respondents (*n* = 839) at an average of 238.7 hrs/month at a mean monthly cost of €2873/person with MS. Informal care was received by 49% of the respondents at an average of 47.3 hrs/month at a mean monthly cost of €389/person with MS. Utilities across disease severity are as follows: mild MS = 0.709 (sd = 0.233), moderate MS = 0.562 (sd = 0.232), and severe MS = 0.284 (sd = 0.283). Total home care costs increased with increasing disease severity. Informal caregiving contributes significantly to MS home care in Sweden.

## 1. Introduction 

In Sweden, estimated 17,000 of the population are diagnosed with multiple sclerosis (MS) [1]. The reported prevalence of MS was 188.9/100,000 of the population in 2008, with twice more women affected than men [2]. About a third of these individuals with MS are over 60 years old. About 5 persons in 100,000 are newly diagnosed with the disease each year.

MS is a chronic, highly variable, and unpredictable disease whose onset may be signaled by an attack of neurologic dysfunction such as blurred vision, extreme fatigue, pain, numbness in extremities, loss of movement, or speech problems (clinically isolated syndrome). This is typically followed by acute relapses in between periods of clinical remission (relapsing-remitting MS). When MS is left untreated, a progressive phase may ensue where there is gradual accumulation of disability with or without relapses (secondary-progressive MS). In about 10% of the diagnosed individuals, neurologic function gradually deteriorates continuously with no occurrence of relapses (primary-progressive MS).

Disease progression is associated with functional disability including mobility problems, bladder dysfunction, pain, depression, and cognitive changes [3]. These impact the patient's ability to do day-to-day activities and increase the reliance on external help [4, 5]. Diseased individuals and people around them need to adapt their lifestyle and/or their working and living environments to accommodate the changing needs of the MS patient. The impact of the disease is most felt by those diagnosed during the productive years when families and careers are developing [6, 7].

Although there is no cure for MS, several disease-modifying therapies exist to reduce relapse rates, slow disability progression, and modify the overall disease course. Long-term therapy is needed, and adherence to therapy is a big challenge. Low adherence to treatment has been shown to lead to higher rates of relapses and progression with its associated increase in the utilization of health care resources [8].

MS results in a considerable economic burden to the Swedish society. Estimates in 2008 euros showed that the total annual costs of MS according to disease severity were the following: EDSS < 4 €27,570, EDSS 4–6.5 €45,769, and EDSS > 6.5 €104,492 with an average total cost per patient amounting to €46,289 [9]. Direct nonmedical costs, that is, formal help in home, transportation, aids, devices, and adaptations, accounted for 30% and informal care accounted for 10% of the total cost per patient [6, 9].

## 2. Objectives

The objectives of this study are to describe and to estimate the costs of formal and informal home care related to MS and the quality of life of the study population.

## 3. Methods

### 3.1. Study Design

A random sample of 1500 members of the Swedish Organization for Patients with Neurological Diseases (Neuroförbundet), specifically those with MS, were mailed a letter with study information and study consent to participate along with the study questionnaire between February and March 2012. The recall period was one month up to the time of the survey. Completed questionnaires containing no patient identification data were then sent back for further analyses. Reminders were sent to all after two weeks.

### 3.2. Data Collection

Respondents were asked to indicate their use of formal and/or informal home caregivers at the time of the study using questions with yes/no answers followed by specification of the type and quantity of the resources used. Instructions to include only MS-related care were given. Formal care data were reported as the number of hours per month that care was performed by publicly funded home help services and/or personal assistance. Informal care data were reported as the number of hours per month that family, relatives, and/or friends help with personal care (bathing, dressing, and moving indoors/outdoors), services (cleaning, washing, and shopping), and transport (travel to/from work and medical visits).

Production losses due to informal caregiving were reported as sick leave days during the last year as well as changes in working hours to accommodate caregiving tasks.

EQ-5D-3L was incorporated in the questionnaire as a measure of health-related quality of life. Respondent characteristics including gender, age, educational level, living situation, and drug treatment received were collected. Disease information included self-reported information on year of MS diagnosis, type of MS, and self-assessed current EDSS level. The Expanded Disability Status Scale (EDSS) measures an individual's functional capacity. In this study, a self-assessed version developed for use in a mail survey was utilized [6].

### 3.3. Costs

Unit costs of formal care were taken from a 2011 report of the Swedish Association of Local Authorities and Regions, where estimates for home help services were calculated and allocated per elderly in 2010. One hour of formal home help services included personnel time in a person's home, travel time, and time for the administration of any treatment. The same unit costs were used for one hour of personal assistance.

Informal care was valued by the opportunity cost approach arguing that time spent on caring cannot be used for other activities such as paid work and/or leisure activities. An hour of informal care was valued by an individual's income loss based on the mean salary (adjusted by average work time) in the general population for 2011 after 32% tax. The production losses of caregivers due to sick leave or changed working hours were valued using paid average wages including the employers' costs of benefit package of 42%. All costs were presented in euros at 2012 prices after adjustments using the consumer price index. Unit costs are presented in Swedish kronor (SEK) and euros, using the annual average 2012 exchange rate of 1 € = 8.7053 SEK (Table 1) [10].

### 3.4. Analysis

Prior to analysis, invalid and inconsistent entries were identified and labeled as missing. To derive the total costs of formal and informal home care, resource utilization data were multiplied by their unit costs and estimated as the mean cost per person with MS. It was assumed that the reported resource utilization during the study represented a typical monthly level of resource use of an individual. The estimation of informal health care costs included the actual hours per month given by informal care, the number of days per year reported as sick leave in order to care for the affected family member, and the reported changes in employment (e.g., decrease in time for paid work) in order to care for the sick. Since costs of care are oftentimes dependent on disease severity, the results were analyzed and reported by EDSS level. EDSS scores were categorized into three severity groups: mild (EDSS 0–3), moderate (EDSS 4–6), and severe (EDSS ≥ 6.5) [11].

Factors that may affect the likelihood that an individual with MS will get home care were also evaluated. A semi-logarithmic linear regression analysis using STATA was performed with the following independent variables: age, gender, housing condition, number of years diagnosed with MS, and disease severity. EQ-5D-3L was not included in the regression analysis because of a high correlation with EDSS.

EQ-5D-3L utilities were estimated using UK tariffs [12].

## 4. Results 

### 4.1. Patient Demographics

A total of 839 questionnaires were received (56% response rate) of which 77% of the responders were female. Of the returned questionnaires, 7 respondents explicitly refused participation to the survey. One of the respondents was excluded from the analysis because he/she was under institutional care during the survey. The mean age of the sample was 56 years (sd = 11.8) with 27% of the respondents having 65 years or more. The mean number of years since the diagnosis of MS was 17 years (sd = 11.3) with 39% of the respondents reporting having primary progressive MS and 26% with secondary progressive MS. Around 39% had severe disease (EDSS ≥ 6.5) and 28% reported having mild disease (EDSS 0–3). The majority of the respondents were living with someone (69%) and 41% attained a high level of education (Table 2).

### 4.2. Formal and Informal Care

Table 3 summarizes the proportions of respondents receiving formal home care and informal care, as well as intensity of services received. In this sample, over a quarter of the respondents (27%) reported receiving formal care at home, 14% received municipal home help services, and 17% received personal assistance services. Both forms of community services were received by 17% of the respondents (*n* = 225). A respondent receives an average of 239 hours per month of formal home care. The mean number of hours of formal care is highest for respondents with severe disease (267 hours/month).

Almost half of the respondents (49%) reported being helped at home by family and friends. Help was most needed with daily activities/services (29%), followed by personal care (13%) and transport (5%). The average number of hours of informal home care was 47 hours per month. Respondents with severe MS needed more help (70 hours) than those with less severe MS. It was also reported that 81% of informal caregivers were cohabitants of the person with MS.

Changes in working hours by informal caregivers as a result of caregiving tasks were reported by 3% of the respondents (Table 3). On average, working time decreased to 16 hours/week (sd = 10.8). Informal caregivers took an average of 8.4 days per year as sick leave days in order to care of the person with MS, as reported by 5% of the respondents.

Across the sample, the mean total number of hours of home care per month was 78.3 hours per person (sd = 6.2), of which informal care accounted for 21.3 hours (sd = 1.7). Monthly production losses due to informal care (i.e., sick leave and modifications in working hours) were estimated to be about 2.2 hours per person (sd = 0.5) (data not shown).

### 4.3. Utility

The EQ-5D was completed by 88% of the respondents. The average estimated utility of the sample was estimated at 0.513 (sd = 0.307). Across disease severity, utility was estimated as follows: mild MS = 0.709 (sd = 0.233), moderate MS = 0.562 (sd = 0.232), and severe MS = 0.284 (sd = 0.283). The health utility scores correlated with the EDSS scores (Kruskal-Wallis *P* < 0, 01).

### 4.4. Costs

The estimated monthly costs for formal and informal care per home care user as well as the mean monthly costs per person with MS in the sample are shown in Table 4. The estimated total monthly home care costs per person are €3262 of which €2873 (88%) accounted for formal care costs and €389 for informal care costs. Monthly production losses due to sick leave and/or lower productivity of caregivers accounted for 18% (€69 per person) of the informal care costs. For respondents with severe MS, the estimated total monthly home care costs amounted to €8446, 90% of which is spent on formal care and 10% on informal care.

Table 5 shows the results of the regression analyses of factors influencing total costs of home care and their categories, that is, formal and informal costs. The reported coefficients reflect the percent change in costs, if the independent variable changes by one unit. Total monthly home care costs are significantly higher for those aged 35–44 years old, those with moderate-severe MS, and those who are cohabiting with someone and are higher as the number of years since the diagnosis of MS increases (all other variables are equal). Men have relatively lower total costs than women (−63%). Respondents in the working age category (35–44 yrs) have twice the total costs of respondents aged 18–34 years. Those with moderate and severe disease have a 3-fold and almost 8-fold increase in the total costs compared to those with mild disease. Respondents cohabiting with someone have 70% higher costs than those living alone.

For formal care costs, factors leading to higher costs include severe MS and higher number of years since diagnosis. To live with someone lowers formal care costs by around 157% compared to those living alone (all other variables are equal). Informal care costs are shown to be higher for those between 35 and 44 years old (141% higher), for those with moderate MS (300% increase) and, for those with severe MS (~500% increase) as well as for those cohabiting with someone (350% increase) compared to those who are younger, those with mild disease, and those who live alone, respectively.

## 5. Discussion 

This mail survey aimed to describe and to estimate the costs associated with formal and informal home care for a random sample of members with MS of the Neuroförbundet, an organization of patients with neurological diseases in Sweden. The sample population consisted of 77% females with a mean age of 56 years and almost 70% of whom were cohabiting. Around 40% of the respondents had primary progressive MS, 24% had relapsing remitting MS, and 26% had secondary progressive MS. Mild, moderate, and severe diseases were reported in approximately 30%, 30%, and 40% of the sample, respectively. It was not possible to determine the representativeness of this sample to the Swedish MS population because of the unavailability of data.

In this sample, respondents with severe disease (EDSS ≥ 6.5) were highly likely to require formal care in terms of home help services and/or personal assistance as well as informal care from family or friends. It is estimated that those with severe MS require 267 hours of formal care per month and 70 hours of informal care per month on the average, amounting to about €8446 per person-month. For those with mild disease (EDSS 0–3), informal care is the most resorted to, requiring only an average of 4 hours of formal care and 14 hours of informal care per month, or €63 per person-month.

In 2006, a major European study on the costs and quality of life of people with MS in nine countries including Sweden using a similar methodology as this study was published [6]. Compared to the 2006 Swedish sample, this present study had more individuals with primary progressive MS and who are aged 65 years and above. The reported utilities as measured by EQ-5D were also slightly lower in our study. Although there are differences in the characteristics of the respondents between the two studies, it is notable that the present study reported the same proportion of formal home care services users.

A comparison of the results shows that the number of hours of home help services per month is lesser in our study, 49 hours per user-month versus 27 hours per user-month, respectively. Although the proportions of respondents who required personal assistance are similar in both studies, the present study reports about 70 hours more per user-month. This finding suggests a shift from home help services to personal assistance between the years 2005 and 2012 when the studies were conducted (data not shown.)

The proportion of respondents receiving informal care is lower in our study (57% versus 49%). Information on how informal care is provided is not available in the Berg study. On average, the reported number of hours for informal care-giving is also lower in our study. This might probably be due to differences in how the questions were worded. In the Berg study, it asked the respondents about the number of hours they had assistance from family and friends over the past month. In our study, the respondent was asked to specify the number of hours they received help with services, personal care, and transport from family or friends. It is highly probable that the Berg study included the number of hours that the family member was present in the home whether or not any service was actually done. Our study showed the complementary nature of informal care to formal care, whereby if no family or friend can give help, then help is provided by formal care services.

The total costs of home care are very much dependent on disease severity, length of MS diagnosis, and living arrangements. Costs of formal care increase with disease severity, whether number of years diagnosed with MS, and the the respondent is living alone. Costs of informal care also increase with the severity of the disease and whether the respondent is cohabiting or not. Compared to respondents with mild MS, those with moderate MS have threefold the cost of mild MS and those with severe MS have a sevenfold increase in the total costs of home care (all other variables are equal).

Production losses attributable to sick leave or decrease in employment amount to 18% of informal care costs. We believe that this is the first time that productivity losses of caregivers of people with MS in Sweden are reported. Due to the overrepresentation of patients with moderate and severe disease, even possibly patients with progressive course of disease, it is not directly possible to extrapolate costs to the overall MS population in Sweden, nor can the study sample be considered representative of the general MS population.

This study can be considered as describing a sample of persons diagnosed with MS belonging to a specific patient organization. Some adjustments were made to make sure that the number of persons within different severity-of-disease categories will be similar for estimation of costs so the sample may not be a valid cross-section of the distribution of MS patients in Sweden. Nonresponse to the mailed survey is estimated at 44% which could have affected the study results. Resource use data was collected using a recall period of one month. It is believed that heterogeneity exists in the number, personal characteristics, availability, and qualifications of the caregivers which could have some impact on the efficiency of how care is given.

## 6. Conclusion 

Formal care costs accounted for a large proportion of total home care costs despite the fact that a greater proportion of MS patients received informal home care in this study sample. Total home care costs increased with increasing disease severity. Patients cohabiting with an informal caregiver had lower formal care costs but higher informal costs. Family members or informal caregivers save the health care system from additional formal care costs but increase the total costs of care to society because of the caregivers' decreases in productivity or sick-leaves. This highlights the contribution of informal caregiving to the overall home care of MS patients in Sweden thus providing the potential of cost-savings for the health care delivery system. Further research into the relationship between formal and informal home caregiving is necessary to help guide health resource allocation for the care of MS patients in Sweden.

## Figures and Tables

**Table 1:** Unit costs.

Cost item	Cost in SEK	Cost in euro	Source
Community services			
Home help services (per hour)	439	50	Swedish Association of Local Authorities and Regions (2011)
Personal assistant (per hour)	439	50	Swedish Association of Local Authorities and Regions (2011)
Informal care (per hour)	146	18	SCB (2012)^1^
Caregivers production losses (per hour)	271	31	SCB (2012)^1;2^
Caregivers sick leave (per day)	1 092	125	SCB (2012)^1;3^

^1^Statistics Sweden (2012) Gross Wages year 2011 (http://www.scb.se/Pages/TableAndChart____149087.aspx).

^2^Assumption: 229 work days per year (full-time) and eight hours per day.

^3^Assumption: full-time wages adjusted for 80% of the employment. Number of sick days was specified in the questionnaire for periods of weeks, where annual earnings were allocated to 365 days.

**Table 2:** Sample demographics.

	Number	Percent
Total number of responders	831	
Gender		
Female	637	77%
Male	189	23%
Missing	5	1%
Age		
18–34 years	34	4%
35–44 years	100	12%
45–54 years	215	26%
55–64 years	247	30%
65-years	227	27%
Missing	8	1%
Education		
Primary school	165	20%
High school degree	319	38%
Professional diploma/university degree	340	41%
Missing	7	1%
Household		
Single	256	31%
Cohabiting	575	69%
Type of MS		
Relapsing-remitting	204	24%
Secondary progressive	220	26%
Primary progressive MS	321	39%
Do not know	76	9%
Missing	10	1%
Using pharmaceutical		
Yes, injection	204	24%
Yes, infusion	83	10%
Yes, oral	109	13%
Yes, combination therapy	12	1%
No	417	50%
Missing	6	1%
Disease severity		
Mild (EDSS 0–3)	236	28%
Moderate (EDSS 4–6)	244	29%
Severe (EDSS ≥ 6.5)	321	39%
Missing	30	4%

**Table 3:** Formal and informal care per home care user.

	Proportion with help	Quantity per home care user during recall period	
	%	Mean	95% CI
*Formal care *	Hours per month		
(Home help services and/or personal assistance)	27%	238.7	203.9–243.5
By disease severity			
Mild (EDSS 0–3)	1%	4	—
Moderate (EDSS 4–6)	9%	38	−6.0–82.0
Severe (EDSS ≥ 6.5)	60%	267	228.4–304.8

*Informal care *	49%	47.3	
Type			
Service		29.0	
Personal care		12.9	
Transport		5.4	
By disease severity			
Mild (EDSS 0–3)	19%	14	11.5–17.6
Moderate (EDSS 4–6)	55%	27	22.2–31.9
Severe (EDSS ≥ 6.5)	68%	70	57.1–83.3

	Days per year		
Caregivers sick leave	5%	8.4	6.3–10.6

	Hours per week		
Caregivers changed working hours	3%	15.8	11.6–20.0

**Table 4:** Estimated monthly costs for formal and informal care per home care user and per person with MS in the sample (euro, 2012 prices).

	Monthly cost per home care user*			Monthly cost per person with MS** (*n* = 740)		
	Mean	95% CI		Mean	95% CI	
*Formal care *						
(Home help services and/or personal assistant)	12 037	10 282	13 791	2 873	2 299	3448
By disease severity					
Mild (EDSS 0–3)	202	0	0	1	−1	3
Moderate (EDSS 4–6)	1 918	−300	4 137	184	−36	403
Severe (EDSS ≥ 6.5)	13 443	11 516	15 369	7 641	6 227	9 055
*Informal care *	893	759	1 026	389	327	451
By disease severity						
Mild (EDSS 0–3)	345	213	478	62	33	91
Moderate (EDSS 4–6)	527	412	643	278	208	347
Severe (EDSS ≥ 6.5)	1 302	1 066	1 538	805	653	957
*Total home care *	5 640	4 688	6 593	3 262	2 677	3 848
By disease severity						
Mild (EDSS 0–3)	342	66	212	63	0	0
Moderate (EDSS 4–6)	791	408	1 174	461	34	92
Severe (EDSS ≥ 6.5)	9 204	7 696	10 712	8 446	233	689

*Estimated for those who availed of formal and/or informal care in sample.

**Estimated for all respondents in the sample, whether had home care or not.

**Table 5:** Results of regression analyses for total home care costs, formal care costs, and informal care costs for persons with MS in Sweden.

Independent variable	Total home care costs (*n* = 699)	Formal care costs (*n* = 747)	Informal care costs (*n* = 718)
	Coefficient	Coefficient	Coefficient
Gender			
Female^†^			
Male	−0.629*	0.316	−0.497
Age in years			
18–34^†^			
35–44	1.824*	0.330	1.412*
45–54	0.508	−0.475	0.227
55–64	0.637	−0.883	0.577
65+	0.408	−1.040	0.327
Disease severity			
Mild (EDSS 1–3)^†^			
Moderate (EDSS 4–5)	3.300*	0.596	2.970*
Severe (EDSS > 6.5)	7.693*	5.395*	4.842*
Living arrangement			
Living alone^†^			
Cohabiting	0.693*	−1.572*	3.512*

Years since MS diagnosis	0.056*	0.091*	−0.002

Constant	−0.455	0.511	−1.703*

*R* ^2^	49.66%	43.41%	33.52%

*P < 0.05; ^†^reference group.
